# Identification of alterations associated with age in the clustering structure of functional brain networks

**DOI:** 10.1371/journal.pone.0195906

**Published:** 2018-05-24

**Authors:** Grover E. C. Guzman, Joao R. Sato, Maciel C. Vidal, Andre Fujita

**Affiliations:** 1 Department of Computer Science, Institute of Mathematics and Statistics, University of São Paulo, São Paulo, Brazil; 2 Center of Mathematics, Computation, and Cognition, Universidade Federal do ABC, Santo André, São Paulo, Brazil; University of Texas at Austin, UNITED STATES

## Abstract

Initial studies using resting-state functional magnetic resonance imaging on the trajectories of the brain network from childhood to adulthood found evidence of functional integration and segregation over time. The comprehension of how healthy individuals’ functional integration and segregation occur is crucial to enhance our understanding of possible deviations that may lead to brain disorders. Recent approaches have focused on the framework wherein the functional brain network is organized into spatially distributed modules that have been associated with specific cognitive functions. Here, we tested the hypothesis that the clustering structure of brain networks evolves during development. To address this hypothesis, we defined a measure of how well a brain region is clustered (network fitness index), and developed a method to evaluate its association with age. Then, we applied this method to a functional magnetic resonance imaging data set composed of 397 males under 31 years of age collected as part of the Autism Brain Imaging Data Exchange Consortium. As results, we identified two brain regions for which the clustering change over time, namely, the left middle temporal gyrus and the left putamen. Since the network fitness index is associated with both integration and segregation, our finding suggests that the identified brain region plays a role in the development of brain systems.

## Introduction

The transition from childhood to adulthood involves major changes in cognitive and emotional functions. These changes are driven by continuous dynamic interactions between genetic profiles and environmental factors, which impact the structural and functional brain networks [1–3]. Current evidence suggests that such neurodevelopmental changes are the result of synaptic pruning and myelination [4–6]. Investigating developmental effects on functional networks is fundamental to better understanding cognition maturation, the hierarchical structure of neural circuitries [2], and the association between neural substrates and many psychiatric and neurological disorders [7–10].

In several neuroimaging studies, the human brain has been generally modeled as a functional network (graph) composed of regions of interest (vertices) and their connections (edges). Pioneering studies based on resting-state protocol and functional magnetic resonance imaging (fMRI) investigated the trajectories of brain networks from childhood to adulthood and identified evidence of an age-evolving functional integration and segregation [11, 12]. These studies found that during the developmental process, the organization of functional networks evolves from a local to a distributed architecture, mainly with regard to long-range connections [7, 12]. In addition, other studies have suggested that children present stronger subcortical-cortical and weaker cortical-cortical connectivity than young adults do [1, 13]. As concluded in a review written by [14], who combined results from [12, 13, 15, 16], there is evidence of greater functional segregation in childhood and increased integration in adulthood.

Recent approaches have focused on the hypothesis that the functional brain network is organized into spatially distributed clusters (sets of vertices that are more tightly connected within a module and less connected to the vertices of other modules) that are associated with specific cognitive functions [17–19]. Some examples of these modules are the visual, default mode, cognitive control, motor, and auditory systems, usually identifiable using resting-state fMRI protocols in groups of subjects.

Several studies have suggested that this modular structure evolves across the life span [20, 21]. For example, [15, 22, 23] investigated the influence of typical trajectories on the modular structure of brain networks. [22] focused on adulthood (from 25 to 65 years of age) while [15] focused on adolescence and early adulthood (from 7 to 31 years of age). [20, 21, 24, 25] identified regions with linear and quadratic trajectories from childhood to adulthood by separately analyzing intra- and inter-modular connectivity. In a study based on a sample of 780 American youths (8 to 22 years old), [25] have demonstrated that brain functional connectivity is subject to a process of modular evolution, balancing within- and between-module connections. This study also showed that individual variability predicts cognitive performance. Moreover, in a review study of functional connectivity in resting-state fMRI, [26] concluded that developmental changes occur through segregation processes of local regions and integration of distant regions from distinct brain modules.

However, since there is evidence that integration and segregation processes are age dependent, the relevance of a brain region to each functional module could be different in childhood, adolescence, and adulthood. As a hypothetical example, one region could present an increased/decreased integration with a module when the individual gets older. However, studies exploring the age dependence of the goodness-of-fit of brain regions into a functional cluster are, to the best of our knowledge, scarce to nonexistent. One challenge in addressing this point is the lack of a proper methodological framework to analyze the data through this cluster analysis perspective. Most studies describe developmental changes in integration/segregation by using seed-based or independent components analyses.

In the current study, we tested the hypothesis that some brain regions change in terms of clustering structure across neurodevelopment (thus, an alternative manner to analyze segregation/integration during development). To investigate this hypothesis, we: (i) first defined an index that measures how well one region of interest (ROI) is clustered (i.e., a network fitness index) in its respective sub-system; and (ii) we identified the ROIs in which this index is associated with age. The intuitive idea is that, if the network fitness index of a region is altered across neurodevelopment, then this region presents an altered connectivity within its module (segregation) and also between regions belonging to other modules (integration). Finally, we applied this method to a large fMRI dataset composed of 397 males under 31 years of age (6.47–30.78 years) collected from the Autism Brain Imaging Data Exchange (ABIDE) Consortium.

## Materials and methods

The data pre-processing, the construction of functional networks, and the clustering approach used here are similar as those described in our previous studies [27, 28] using the ABIDE dataset. We briefly describe them in the following sub-sections.

### Data description and pre-processing

An fMRI dataset composed of 1112 individuals was downloaded from the ABIDE Consortium website (http://fcon_1000.projects.nitrc.org/indi/abide/). The research performed at the ABIDE contributing sites complied with Health Insurance Portability and Accountability Act (HIPAA) guidelines and the 1000 Functional Connectomes Project/International Data-sharing Initiative protocols. All data distributed via the ABIDE website were fully anonymized in compliance with the HIPAA privacy rules, and no protected health information was included. To study how the clustering structure evolves over development, we excluded 367 individuals diagnosed with autism spectrum disorder (ASD), 57 without ASD subtype classification, all the 141 females, and 33 older than 31. To pre-process the fMRI data, we used the Athena pipeline (http://www.nitrc.org/plugins/mwiki/index.php/neurobureau:AthenaPipeline), which excluded 10 subjects. To minimize the effects of head movement during magnetic resonance scanning, we used the “scrubbing” procedure proposed by [29] and used in several other works [30–33]. Volumes with frame-wise displacement (FD) or temporal derivative of the root mean square (RMS) variance larger than the 95% percentile were excluded (two individuals). One hundred and five individuals with less than 100 volumes after scrubbing were also excluded. Thus, the final dataset used in our analysis was composed of 397 males (mean age ± standard deviation, 16.29 ± 5.61 years) distributed along 19 datasets collected from 16 international sites (there is more than one dataset per site) of the ABIDE Consortium (Table 1). All data collection was conducted with local internal review board approval and in accordance with local internal review board protocols. For further details about this dataset, refer to the ABIDE Consortium website.

**Table 1:** Demographic summaries for all datasets provided by the ABIDE Consortium. SD: standard deviation.

Dataset	Site	Male		
		*n*	age (mean ± SD)	range
1	Caltech	9	21.89 ± 3.26	17.00–27.90
2	CMU	7	23.86 ± 2.79	21.00–27.00
3	KKI	21	10.23 ± 1.37	8.39–12.77
4	Leuven	15	23.27 ± 2.91	18.00–29.00
5	Leuven	15	14.60 ± 1.62	12.20–16.90
6	MaxMun	18	22.06 ± 6.86	7.00–30.00
7	NYU	78	15.75 ± 5.99	6.47–30.78
8	Olin	14	16.86 ± 3.82	10.00–23.00
9	Pitt	21	18.05 ± 5.70	9.44–30.66
10	SBL	5	25.40 ± 3.05	20.00–27.00
11	SDSU	15	14.47 ± 1.35	12.58–16.60
12	Stanford	16	10.19 ± 1.66	8.25–12.43
13	Trinity	23	17.48 ± 3.66	12.04–25.66
14	UCLA	19	13.41 ± 1.99	9.50–17.78
15	UCLA	5	12.28 ± 1.54	9.79–13.63
16	UM	38	13.73 ± 3.28	8.20–18.90
17	UM	21	16.73 ± 3.96	13.30–28.80
18	USM	37	19.78 ± 5.62	9.92–29.97
19	Yale	20	12.34 ± 2.79	7.66–17.83

### Functional connectivity networks

To define the regions of interest (ROIs), we used the CC400 atlas [34]. Thirty-five ROIs including the ventricles were identified by using the MNI atlas and removed, leaving 316 ROIs. The average time-series signal of all voxels belonging to a ROI was considered to be representative of the region. The functional brain network for each subject was constructed by estimating the Pearson’s correlation coefficient among the 316 ROIs (i.e., the similarity matrices **M**_*t*_ for *t* = 1, …, 397), and the site effects (different fMRI recording sites) were removed using a generalized linear model with the Pearson’s correlation coefficient as the response variable and the site as an independent covariate. Residues of this model were considered to be the Pearson’s correlation coefficients without the site effect. Then, we discarded negative correlations as described by [12, 31, 35–37]. Finally, the average functional network ($\bar{M}$) was constructed by calculating the average of the Pearson’s correlation coefficients (covariated by site). The average functional network $\bar{M}$ will be used in Clustering analysis section to obtain a reference clustering structure.

### Silhouette statistic

The silhouette statistic was proposed in 1987 by [38] to estimate the number of clusters in a dataset. The interpretation of this statistic is that it quantifies how well a specific item is fitted to the cluster it was assigned to by the clustering algorithm. In other words, the silhouette statistic is a measure of the goodness-of-fit of the obtained clustering structure. Thus, the number of clusters that maximize the silhouette statistic is the suggested number of modules in the dataset.

The silhouette statistic can be formalized as follows. Let *X* = {*x*_1_, …, *x*_*n*_} be the ROIs that are clustered into *C* = {*C*_1_, …, *C*_*k*_} clusters (sub-networks) by the spectral clustering algorithm ($X=\cup_{q=1}^{k}C_{q}$) [39]. Let *d*(*x*, *y*) be the dissimilarity between ROIs *x* and *y* (one minus the Pearson’s correlation coefficient between *x* and *y*), and let |*C*| be the number of ROIs of *C*. Then, we define the average dissimilarity of *x* to all ROIs of cluster *C* ⊂ *X* as follows:

$$\begin{array}{c} d\left(x,C\right)=\frac{1}{\left|C\right|}\sum_{y\in C}^{}d\left(x,y\right). \end{array}$$ (1)

Denote by *D*_*q*_ ∈ *C* the cluster to which *x*_*q*_ has been assigned by the clustering algorithm and by *E*_*q*_ ∈ *C* any other cluster different from *D*_*q*_, for all *q* = 1, …, *n*. Let *a*_*q*_ be the dissimilarity of *x*_*q*_ to the cluster to which it was assigned to and *b*_*q*_ be the minimum dissimilarity of *x*_*q*_ among all other clusters different from the cluster that it was assigned to by the algorithm. In other words, we have the following:

$$\begin{array}{c} a_{q}=d\left(x_{q},D_{q}\right)\;\text{and}\;b_{q}=\min_{E_{q}\neq D_{q}}d\left(x_{q},E_{q}\right),\;\text{for}\;q=1,\ldots ,n. \end{array}$$ (2)

Then, the silhouette statistic is defined as:

$$\begin{array}{c} S_{q}=\{\begin{array}{cc} \frac{b_{q}-a_{q}}{max\{b_{q},a_{q}\}} & \;\text{if}\;|D_{q}|>1\\ 0 & \;\text{if}\;|D_{q}|=1 \end{array} \end{array}$$ (3)

The interpretation of the silhouette statistic is as follows [38]: if *s*_*q*_ ≈ 1, then *a*_*q*_ ≪ *b*_*q*_, i.e., the distance of ROI *x*_*q*_ to the cluster it was assigned to is smaller than the smallest dissimilarity between *x*_*q*_ and other clusters. In other words, the ROI *x*_*q*_ has been assigned to an appropriate cluster because the second-best choice cluster is not as close as the actual cluster. If *s*_*q*_ ≈ −1, it means that *a*_*q*_ ≫ *b*_*q*_, i.e., the ROI *x*_*q*_ lies much closer to the second-best choice cluster than to the cluster that this item was assigned to by the clustering algorithm. Hence, it is more natural to assign the ROI *x*_*q*_ to the second-best choice cluster than to the actual cluster because this ROI *x*_*q*_ has been “misclassified”. Finally, if *s*_*q*_ ≈ 0, then *a*_*q*_ ≈ *b*_*q*_; therefore, it is not clear whether *x*_*q*_ should have been assigned to the actual cluster or to the second-best cluster because it lies equally far away from both. Based on these interpretations (goodness-of-fit), we will define the silhouette statistic *s*_*q*_ as the Network Fitness Index (NFI).

### Clustering analysis

To identify the ROIs associated with age in terms of functional clustering, we developed a framework based on the NFI. We describe our problem in the following way: let *n* be the number of ROIs, *t* be the number of subjects, and **M**_1_, …, **M**_*t*_ be (*n* × *n*) similarity matrices representing the functional brain networks, with a column vector **age** = [*age*_1_, …, *age*_*t*_]^⊤^ containing the ages of each subject. We would like to identify the ROIs that cluster in a different manner in accordance with age. To calculate the NFI for each ROI of each subject, it is necessary to apply a common reference clustering structure (the clustering label of the ROIs) to all individuals. Thus, we applied the spectral clustering algorithm [39] on the similarity matrix $\bar{M}$ to obtain the reference *k* sub-networks (clusters) *C* = {*C*_1_, …, *C*_*k*_}. Briefly, to cluster the ROIs into *k* modules, the spectral clustering algorithm selects the *k* eigenvectors associated with the *k* smallest eigenvalues of the similarity matrix $\bar{M}$ and uses them as *k*-dimensional points. Then, the k-means clustering algorithm is applied to these *k*-dimensional points. Here, rather than using the usual k-means algorithm inside the spectral clustering, we used the k-medoids algorithm because the latter is more robust to outliers than the former [40]. Similar to other clustering algorithms, the spectral clustering algorithm also requires the number of clusters *k* as an input. We estimated the number of clusters by using the silhouette criterion, i.e., the number of clusters that maximizes the silhouette statistic [38].

### Identification of brain regions associated with age

**Fig 1:**
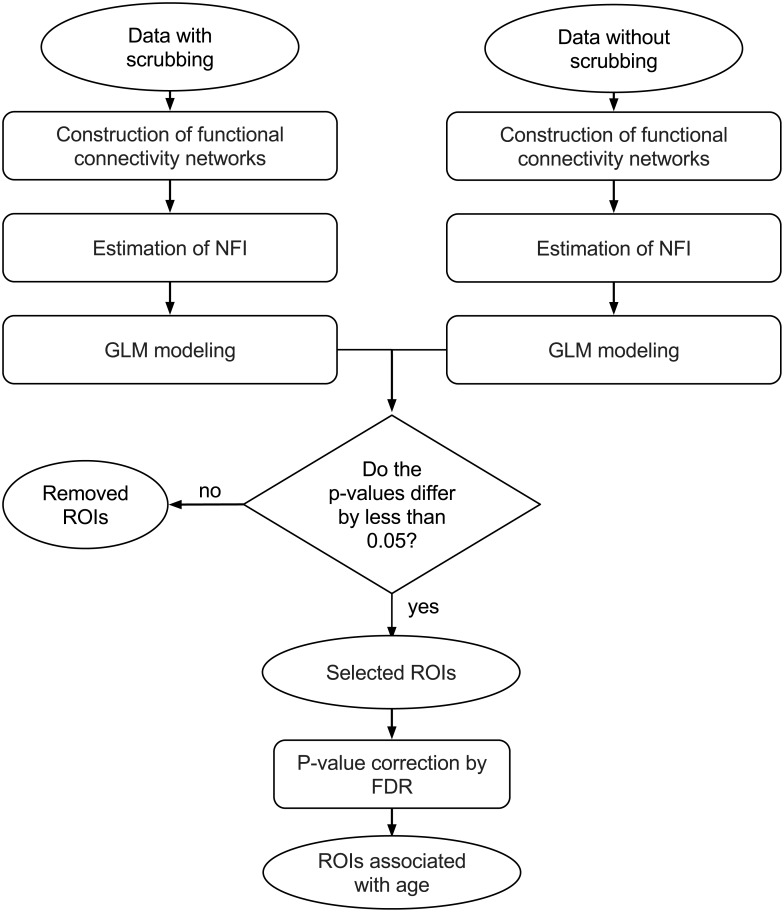
Data analysis pipeline. First, functional connectivity (FC) networks were constructed by estimating the Pearson’s correlation coefficient between ROIs. Second, the spectral clustering algorithm was applied to the average functional network to obtain the sub-networks (clusters). Then, the network fitness index (NFI) was calculated for each ROI. To identify the ROIs associated with age, we modeled the NFI using a generalized linear model with the number of volumes removed by the “scrubbing” procedure as a covariate. This procedure was applied to data with and without “scrubbing” per site. P-values of different sites were combined by using the Fisher’s method. ROIs that presented a difference in nominal p-values greater than 5% were excluded. The remaining p-values were corrected for false discovery rate. ROIs with a corrected p-value lower than 5% in the scrubbed data were considered to be associated with age.

The identification of ROIs associated with age regarding their clustering structure was performed using a generalized linear model (GLM) with the NFI as the response variable and age as the predictor variable in each site with more than 30 subjects separately (because the ABIDE data set is known to present site effects, i.e., different scanners and acquisition protocols). To remove the effect of the proportion of volumes removed by the “scrubbing” procedure, we included it as a covariate. To combine the p-values obtained from the different sites we carried out the Fisher’s method (meta analysis). ROIs presenting differences in nominal p-values greater than 5% between results obtained in data with and without “scrubbing” were discarded. A p-value threshold of 5% after correction for multiple tests by the false discovery rate (FDR) procedure [41] on data with “‘scrubbing” was considered to be statistically significant. The whole pipeline is summarized in Fig 1.

To further understand how the clustering structure changes at different neurodevelopmental stages, we divided the data into three groups, namely, children (subjects with ages between 6 and 13), adolescents (subjects with ages between 13 and 18), and adults (subjects with ages between 18 and 31): (i) 56 children (mean age ± standard deviation, 10.59 ± 1.61), (ii) 46 adolescents (15.72 ± 1.28), and (iii) 51 adults (22.85 ± 3.94).

## Results

**Fig 2:**
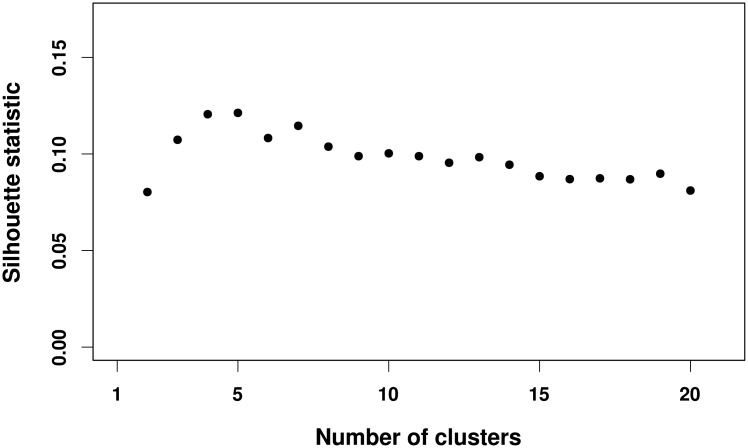
Application of the silhouette statistic to estimate the number of sub-networks. The *x* and *y* axes represent the number of clusters and the silhouette statistic, respectively. Note that the largest silhouette statistic is obtained when the number of clusters is equal to five. In other words, the silhouette statistic suggests clustering the brain functional network into five sub-networks.

**Fig 3:**
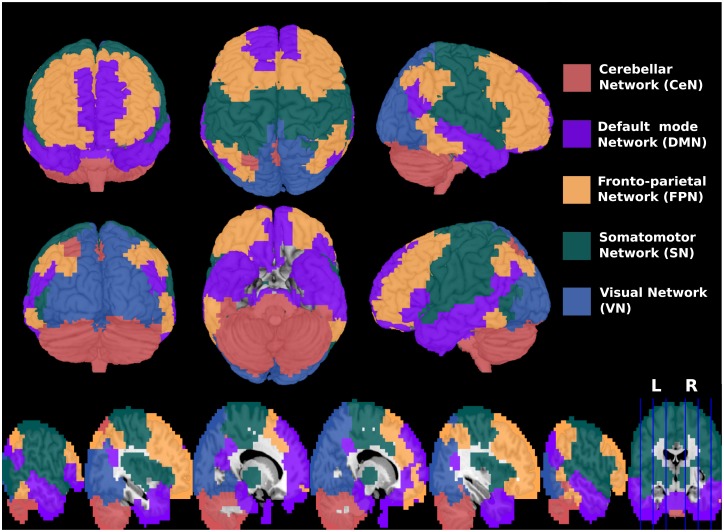
Three-dimensional views and sagittal slices of the entire brain. The five sub-networks obtained by the spectral clustering algorithm, namely, cerebellar, default mode, fronto-parietal, somatomotor, and visual, are discriminated by different colors. L: Left; R: Right.

We pre-processed the fMRI data and constructed the functional brain networks as described in the “Materials and Methods” section. To select the number of clusters, we used the silhouette criterion [38]. Note that the highest value for the silhouette statistic is obtained when the number of clusters is *k* = 5 (Fig 2). Therefore, we set the number of clusters to *k* = 5, which is the estimated value by the silhouette statistic and also consistent with other reports [18, 19, 42]. The five sub-networks identified by the spectral clustering algorithm [39] are illustrated using different colors in Fig 3, namely the cerebellar network (CeN), default mode network (DMN), fronto-parietal network (FPN), somatomotor network (SN), and visual network (VN). We also analyzed when the number of clusters is set to *k* = 4 due to the similar silhouette statistic. In this case, the default mode and fronto-parietal networks become only one cluster, and the cerebellar system included part of the default mode network (S1 Fig). Thus, we considered *k* = 5 for further analyses.

Then, the identification of the ROIs associated with age was performed on data with and without “scrubbing”. Eighty-five ROIs presenting a difference in nominal p-values greater than 5% between the analysis of data with and without “scrubbing” were excluded (high differences in p-values with and without scrubbing means that they are highly affected by head movement). Finally, the p-values for the remaining 231 ROIs were corrected for multiple tests using the FDR procedure [41]. ROIs with *p* < 0.05 (after correction) were considered as statistically significant.

**Fig 4:**
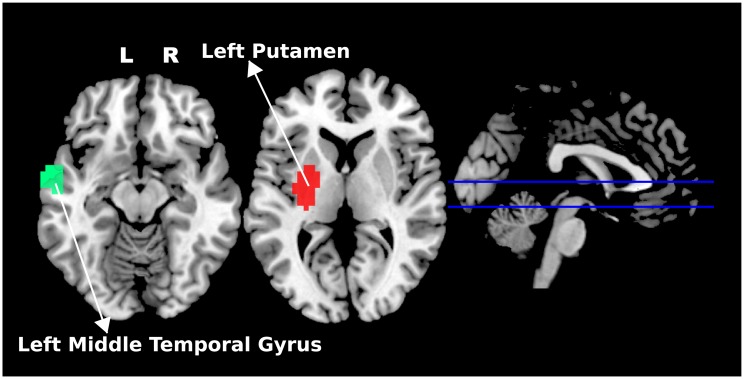
ROIs associated with age. The left middle temporal gyrus and the left putamen are highlighted in different colors. L: left; R: right.

**Fig 5:**
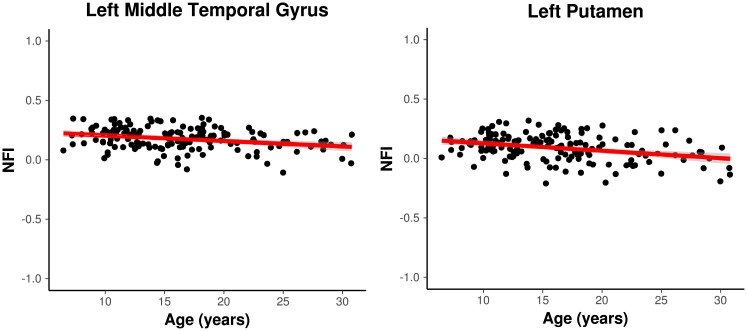
Fitted line obtained for the left middle temporal gyrus and the left putamen. The *x* and *y* axes represent the age in years and the network fitness index (NFI), respectively. Note that the NFIs are negatively associated with age in both ROIs. The slopes are −0.005 with *p* < 0.001 for the left middle temporal gyrus and −0.007 with *p* < 0.001 for the left putamen.

Only two ROIS presented clustering goodness-of-fit associated with age, namely the left middle temporal gyrus and the left putamen (Fig 4). Fig 5 illustrates the associations between age and the network fitness index (NFI—a measure of goodness-of-fit of the ROI in the cluster) for both ROIs. Note that the NFI is negatively associated with age (the slopes are −0.005 with *p* < 0.001 for the left middle temporal gyrus and −0.007 with *p* < 0.001 for the left putamen). In other words, both ROIs tend to integrate with other brain systems (clusters) from childhood to adulthood.

**Fig 6:**
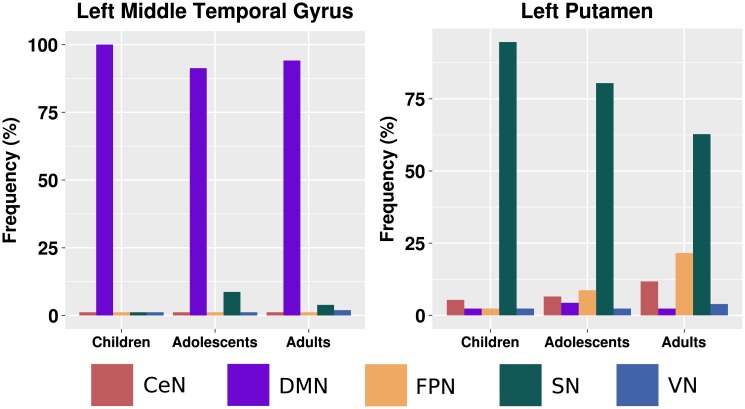
The proportion of subjects that present the left middle temporal gyrus and the left putamen assigned to a specific cluster in each neurodevelopmental stage (children, adolescents, and adults). CeN: cerebellar network; DMN: default mode network; FPN: fronto-parietal network; SN: somatomotor network; and VN: visual network.

Additionally, we calculated the proportion of subjects assigned to each brain module in each neurodevelopmental stage (children, adolescents, and adults) (Fig 6). By analyzing Fig 6, it is clear that the left putamen gradually diverges from the sensorimotor network to become more integrated with the cerebellar system during development. Additionally, after childhood, the left middle temporal gyrus starts diverging from the default mode network to also participate in other systems, particularly, the somatomotor network.

**Fig 7:**
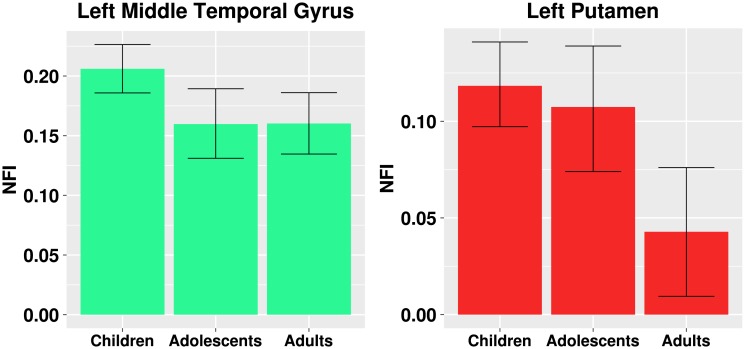
The average and 95% confidence intervals (represented by error bars) of the network fitness index for the left middle temporal gyrus and the left putamen in each developmental stage. The error bars represent the 95% confidence intervals.

Fig 7 shows how the NFI changes over each developmental stage (childhood, adolescence, adulthood). For the left putamen, in fact the NFI decreases as the age increases. In other words, it corroborates the results obtained in Figs 4 and 5 that the left putamen is clustered in a different manner along neurodevelopment (and not in a specific transition between two stages). Furthermore, the same effect is observed in the left middle temporal gyrus.

To certify that these results are not due to numerical fluctuations or another source of error that was not taken into account, we verified the control of the type I error. The ages of the individuals were permuted and the association between the NFI and age re-calculated for the left middle temporal gyrus and left putamen. This procedure was repeated 1000 times. The proportion of falsely rejected null hypothesis for p-values lower than 1, 5, and 10% were 1.1, 3.7, and 10%, respectively for the left middle temporal gyrus and 0.5, 4.5, and 10.7%, respectively for the left putamen. Therefore, these results confirm that the type I error is effectively controlled in this data set.

To exclude the possibility that our results are due to unbalanced data (the number of individuals in the three sites used to estimate the association between NFI and age are 56 children, 46 adolescents, and 51 adults, i.e., a lower number of adults when compared to the set children + adolescents), we carried out the following experiment. We randomly re-sampled (with replacement) the same number of adults and children + adolescents, i.e., 51 adults and 51 from the set children + adolescents. Then, we performed the regression between NFI and age. We repeated this procedure 100 times and estimated the slope and respective p-value for the left middle temporal gyrus and the left putamen. The number of times that the slope’s p-values were lower than 5, 1, and 0.1% were 99, 91, 49 for the left middle temporal gyrus, and 100, 96, 59 for the left putamen. Therefore, these results suggest that the significant relationships between NFI and age in these two ROIs are not due to unbalanced data.

## Discussion

In the current study, we performed a whole brain functional network analysis to identify regions that are differentially clustered among childhood, adolescence, and adulthood. Functional connectivity was identified by using the Pearson’s correlation coefficient. We defined the sub-networks by applying the spectral clustering algorithm, namely, the cerebellar network (CeN), default mode network (DMN), fronto-parietal network (FPN), somatomotor network (SN), and visual network (VN). These five modules are very similar to those that we identified in our previous studies [27, 28] using other subsets of the ABIDE dataset.

The left putamen (Fig 4) identified as exhibiting significant age effects on clustering network fitness index (NFI) presented a reduction in the NFI over development (Fig 4), suggesting that this region may function as inter-system bridge with age. An increase in the inter-modular connectivity could be explained by the increasing connections between other modules through myelination of the brain pathways [43]. The putamen is well established as an associative region. Thus, it is reasonable to expect developmental effects on this region, mainly regarding its role as integrative region in the overall brain network. The putamen, an element of the basal ganglia, is a hub that connects many systems, and it is involved in several functions, such as motor control [44], learning [45], and motivational outcomes of action [46]. Previous studies have investigated developmental effects on functional integration and segregation [14] by using centrality measures. However, none of them were based on the analysis of the evaluation of the quality of modular organization (i.e., clustering goodness-of-fit). Fig 6 suggests that during development, the left putamen becomes more integrated with the cerebellum. To the best of our knowledge, this is the first study to report this finding. Notably, it is well established in the literature that the putamen plays different roles in motor control [47].

Similarly, the left middle temporal gyrus, an associative region well established in cognitive processes involving memory, was also found to present a reduction in NFI. This finding points out that this region plays a role in integrating the DMN and other subsystems. However, further studies are necessary to understand the role of temporal regions in the dynamics of other brain systems, but this result is in agreement with previous neurodevelopmental studies reporting that DMN organization changes during childhood establishing long-range connections [7, 12, 15].

As limitations of the current study, we first note that head movement during fMRI scanning may affect functional connectivity estimates in developmental studies [29, 48] by increasing short-distance correlations (between blood oxygen level-dependent signals) and decreasing long-distance correlations. Thus, to minimize the effects of head movement in our analyses, we applied the “scrubbing” procedure proposed by [29] and adopted a conservative approach, i.e., we discarded any ROI with difference in p-values greater than 5% between data with and without scrubbing. Since this is a developmental sample, we preferred to discard any findings, which could potentially be altered by scrubbing. Moreover, we included the proportion of scrubbed volumes in each subject as a covariate in the GLM model. The rationale is that individual realignment at preprocessing and scrubbing may not be completely eliminated due to non-linear artifactual components. It is important to remark that data scrubbing impacts the p-values associated with functional connections, by reducing the evidence against the null hypothesis. Thus, our approach based on p-values instead of the correlation coefficient is conservative. Second, note that the dataset is cross-sectional, whereas longitudinal data would be more appropriate for determining neurodevelopmental trajectories and also to identify the subgroup of subjects that presents the characteristics observed here. However, cross-sectional data are sufficient to demonstrate age effects on brain organization. Finally, the data were collected from 16 different sites, which may have used different scanners and protocols. For these reasons, we analyzed each site independently and also included the site as a covariate in GLM to minimize its effects.

## Conclusion

In conclusion, we identified two regions with NFI associated with age and that maybe more integrated with other brain system, perhaps playing the role as a bridge between multiple systems. However, additional studies based on diffusion imaging and/or histology are still necessary. Finally, it is important to mention that defining typical developing age effects is a crucial first step to identifying deviant patterns that may lead to future mental disorders [49]. Possible future studies would be the investigation of divergent patterns in clinical and sub-clinical populations.

## Supporting information

S1 Fig Three-dimensional views of the entire brain when the number of clusters is set to four. The four sub-networks obtained by the spectral clustering algorithm are discriminated by different colors. L: Left; R: Right).
(EPS)

## References

[1] Power JD, Fair DA, Schlaggar BL, Petersen SE. The development of human functional brain networks. Neuron. 2010;67(5):735–748. doi: 10.1016/j.neuron.2010.08.017 20826306 10.1016/j.neuron.2010.08.017 PMC2941973

[2] Sporns O. Networks of the Brain. MIT press; 2011.

[3] Rubia K. Functional brain imaging across development. European child & adolescent psychiatry. 2013;22(12):719–731. doi: 10.1007/s00787-012-0291-8 22729957 10.1007/s00787-012-0291-8 PMC3853580

[4] Gogtay N, Giedd JN, Lusk L, Hayashi KM, Greenstein D, Vaituzis AC, et al Dynamic mapping of human cortical development during childhood through early adulthood. Proceedings of the National Academy of sciences of the United States of America. 2004;101(21):8174–8179. doi: 10.1073/pnas.0402680101 15148381 10.1073/pnas.0402680101 PMC419576

[5] Stiles J, Jernigan TL. The basics of brain development. Neuropsychology review. 2010;20(4):327–348. doi: 10.1007/s11065-010-9148-4 21042938 10.1007/s11065-010-9148-4 PMC2989000

[6] Petanjek Z, Judaš M, Šimić G, Rašin MR, Uylings HB, Rakic P, et al Extraordinary neoteny of synaptic spines in the human prefrontal cortex. Proceedings of the National Academy of Sciences. 2011;108(32):13281–13286. doi: 10.1073/pnas.1105108108 10.1073/pnas.1105108108 PMC3156171 21788513

[7] Sato JR, Salum GA, Gadelha A, Picon FA, Pan PM, Vieira G, et al Age effects on the default mode and control networks in typically developing children. Journal of psychiatric research. 2014;58:89–95. doi: 10.1016/j.jpsychires.2014.07.004 25085608 10.1016/j.jpsychires.2014.07.004

[8] Sato JR, Biazoli CE, Salum GA, Gadelha A, Crossley N, Satterthwaite TD, et al Temporal stability of network centrality in control and default mode networks: Specific associations with externalizing psychopathology in children and adolescents. Human brain mapping. 2015;36(12):4926–4937. doi: 10.1002/hbm.22985 26350757 10.1002/hbm.22985 PMC6868942

[9] Sato JR, Salum GA, Gadelha A, Vieira G, Zugman A, Picon FA, et al Decreased centrality of subcortical regions during the transition to adolescence: a functional connectivity study. Neuroimage. 2015;104:44–51. doi: 10.1016/j.neuroimage.2014.09.063 25290886 10.1016/j.neuroimage.2014.09.063

[10] Sato JR, Salum GA, Gadelha A, Crossley N, Vieira G, Manfro GG, et al Default mode network maturation and psychopathology in children and adolescents. Journal of Child Psychology and Psychiatry. 2016;57(1):55–64. doi: 10.1111/jcpp.12444 10.1111/jcpp.12444 26111611

[11] Dosenbach NU, Fair DA, Miezin FM, Cohen AL, Wenger KK, Dosenbach RA, et al Distinct brain networks for adaptive and stable task control in humans. Proceedings of the National Academy of Sciences. 2007;104(26):11073–11078. doi: 10.1073/pnas.0704320104 10.1073/pnas.0704320104 PMC1904171 17576922

[12] Fair DA, Dosenbach NU, Church JA, Cohen AL, Brahmbhatt S, Miezin FM, et al Development of distinct control networks through segregation and integration. Proceedings of the National Academy of Sciences. 2007;104(33):13507–13512. doi: 10.1073/pnas.0705843104 10.1073/pnas.0705843104 PMC1940033 17679691

[13] Supekar K, Musen M, Menon V. Development of large-scale functional brain networks in children. PLoS Biol. 2009;7(7):e1000157 doi: 10.1371/journal.pbio.1000157 19621066 10.1371/journal.pbio.1000157 PMC2705656

[14] Uddin LQ, Supekar K, Menon V. Typical and atypical development of functional human brain networks: insights from resting-state FMRI. Frontiers in systems neuroscience. 2010;4:21 doi: 10.3389/fnsys.2010.00021 20577585 10.3389/fnsys.2010.00021 PMC2889680

[15] Fair DA, Cohen AL, Power JD, Dosenbach NU, Church JA, Miezin FM, et al Functional brain networks develop from a “local to distributed” organization. PLoS comput biol. 2009;5(5):e1000381 doi: 10.1371/journal.pcbi.1000381 19412534 10.1371/journal.pcbi.1000381 PMC2671306

[16] Kelly AC, Di Martino A, Uddin LQ, Shehzad Z, Gee DG, Reiss PT, et al Development of anterior cingulate functional connectivity from late childhood to early adulthood. Cerebral cortex. 2009;19(3):640–657. doi: 10.1093/cercor/bhn117 18653667 10.1093/cercor/bhn117

[17] Biswal B, Zerrin Yetkin F, Haughton VM, Hyde JS. Functional connectivity in the motor cortex of resting human brain using echo-planar MRI. Magnetic resonance in medicine. 1995;34(4):537–541. doi: 10.1002/mrm.1910340409 8524021 10.1002/mrm.1910340409

[18] Damoiseaux J, Rombouts S, Barkhof F, Scheltens P, Stam C, Smith SM, et al Consistent resting-state networks across healthy subjects. Proceedings of the national academy of sciences. 2006;103(37):13848–13853. doi: 10.1073/pnas.0601417103 10.1073/pnas.0601417103 PMC1564249 16945915

[19] Buckner RL, Krienen FM, Yeo BT. Opportunities and limitations of intrinsic functional connectivity MRI. Nature neuroscience. 2013;16(7):832–837. doi: 10.1038/nn.3423 23799476 10.1038/nn.3423

[20] Cao M, Wang JH, Dai ZJ, Cao XY, Jiang LL, Fan FM, et al Topological organization of the human brain functional connectome across the lifespan. Developmental cognitive neuroscience. 2014;7:76–93. doi: 10.1016/j.dcn.2013.11.004 24333927 10.1016/j.dcn.2013.11.004 PMC6987957

[21] Betzel RF, Byrge L, He Y, Goñi J, Zuo XN, Sporns O. Changes in structural and functional connectivity among resting-state networks across the human lifespan. Neuroimage. 2014;102:345–357. doi: 10.1016/j.neuroimage.2014.07.067 25109530 10.1016/j.neuroimage.2014.07.067

[22] He Y, Wang J, Wang L, Chen ZJ, Yan C, Yang H, et al Uncovering intrinsic modular organization of spontaneous brain activity in humans. PloS one. 2009;4(4):e5226 doi: 10.1371/journal.pone.0005226 19381298 10.1371/journal.pone.0005226 PMC2668183

[23] Meunier D, Achard S, Morcom A, Bullmore E. Age-related changes in modular organization of human brain functional networks. Neuroimage. 2009;44(3):715–723. doi: 10.1016/j.neuroimage.2008.09.062 19027073 10.1016/j.neuroimage.2008.09.062

[24] Tomasi D, Volkow ND. Aging and functional brain networks. Molecular psychiatry. 2012;17(5):549–558. doi: 10.1038/mp.2011.81 10.1038/mp.2011.81 PMC3193908 21727896

[25] Gu S, Satterthwaite TD, Medaglia JD, Yang M, Gur RE, Gur RC, et al Emergence of system roles in normative neurodevelopment. Proceedings of the National Academy of Sciences. 2015;112(44):13681–13686. doi: 10.1073/pnas.1502829112 10.1073/pnas.1502829112 PMC4640772 26483477

[26] Vogel AC, Power JD, Petersen SE, Schlaggar BL. Development of the brain’s functional network architecture. Neuropsychology review. 2010;20(4):362–375. doi: 10.1007/s11065-010-9145-7 20976563 10.1007/s11065-010-9145-7 PMC3811138

[27] Sato JR, Balardin J, Vidal MC, Fujita A. Identification of segregated regions in the functional brain connectome of autistic patients by a combination of fuzzy spectral clustering and entropy analysis. J Psychiatry Neurosci. 2015;1:8872147. 10.1503/jpn.140364 PMC4764481 26505141

[28] Sato JR, Vidal M, de Siqueira Santos S, Massirer KB, Fujita A. Complex network measures in Autism Spectrum Disorders. IEEE/ACM Transactions on Computational Biology and Bioinformatics. 2015;PP(99). 10.1109/TCBB.2015.2476787 26353378

[29] Power JD, Barnes KA, Snyder AZ, Schlaggar BL, Petersen SE. Spurious but systematic correlations in functional connectivity MRI networks arise from subject motion. Neuroimage. 2012;59(3):2142–2154. doi: 10.1016/j.neuroimage.2011.10.018 22019881 10.1016/j.neuroimage.2011.10.018 PMC3254728

[30] Leonardi N, Richiardi J, Gschwind M, Simioni S, Annoni JM, Schluep M, et al Principal components of functional connectivity: a new approach to study dynamic brain connectivity during rest. NeuroImage. 2013;83:937–950. doi: 10.1016/j.neuroimage.2013.07.019 23872496 10.1016/j.neuroimage.2013.07.019

[31] Power JD, Schlaggar BL, Lessov-Schlaggar CN, Petersen SE. Evidence for hubs in human functional brain networks. Neuron. 2013;79(4):798–813. doi: 10.1016/j.neuron.2013.07.035 23972601 10.1016/j.neuron.2013.07.035 PMC3838673

[32] van den Heuvel MP, Sporns O, Collin G, Scheewe T, Mandl RC, Cahn W, et al Abnormal rich club organization and functional brain dynamics in schizophrenia. JAMA psychiatry. 2013;70(8):783–792. doi: 10.1001/jamapsychiatry.2013.1328 23739835 10.1001/jamapsychiatry.2013.1328

[33] Lee MH, Hacker CD, Snyder AZ, Corbetta M, Zhang D, Leuthardt EC, et al Clustering of resting state networks. PloS one. 2012;7(7):e40370 doi: 10.1371/journal.pone.0040370 22792291 10.1371/journal.pone.0040370 PMC3392237

[34] Craddock RC, James GA, Holtzheimer PE, Hu XP, Mayberg HS. A whole brain fMRI atlas generated via spatially constrained spectral clustering. Human brain mapping. 2012;33(8):1914–1928. doi: 10.1002/hbm.21333 21769991 10.1002/hbm.21333 PMC3838923

[35] Power JD, Cohen AL, Nelson SM, Wig GS, Barnes KA, Church JA, et al Functional network organization of the human brain. Neuron. 2011;72(4):665–678. doi: 10.1016/j.neuron.2011.09.006 22099467 10.1016/j.neuron.2011.09.006 PMC3222858

[36] Rubinov M, Sporns O. Complex network measures of brain connectivity: uses and interpretations. Neuroimage. 2010;52(3):1059–1069. doi: 10.1016/j.neuroimage.2009.10.003 19819337 10.1016/j.neuroimage.2009.10.003

[37] Zuo XN, Ehmke R, Mennes M, Imperati D, Castellanos FX, Sporns O, et al Network centrality in the human functional connectome. Cerebral cortex. 2011;22(8):1862–1875. doi: 10.1093/cercor/bhr269 21968567 10.1093/cercor/bhr269

[38] Rousseeuw PJ. Silhouettes: a graphical aid to the interpretation and validation of cluster analysis. Journal of computational and applied mathematics. 1987;20:53–65. doi: 10.1016/0377-0427(87)90125-7

[39] Von Luxburg U. A tutorial on spectral clustering. Statistics and computing. 2007;17(4):395–416. doi: 10.1007/s11222-007-9033-z

[40] Kaufman L, Rousseeuw PJ. Finding groups in data: an introduction to cluster analysis. vol. 344 John Wiley & Sons; 2009.

[41] Benjamini Y, Hochberg Y. Controlling the false discovery rate: a practical and powerful approach to multiple testing. Journal of the Royal Statistical Society Series B (Methodological). 1995; p. 289–300.

[42] De Luca M, Beckmann C, De Stefano N, Matthews P, Smith SM. fMRI resting state networks define distinct modes of long-distance interactions in the human brain. Neuroimage. 2006;29(4):1359–1367. doi: 10.1016/j.neuroimage.2005.08.035 16260155 10.1016/j.neuroimage.2005.08.035

[43] Giedd JN, Blumenthal J, Jeffries NO, Castellanos FX, Liu H, Zijdenbos A, et al Brain development during childhood and adolescence: a longitudinal MRI study. Nature neuroscience. 1999;2(10):861–863. doi: 10.1038/13158 10491603 10.1038/13158

[44] Turner RS, Desmurget M, Grethe J, Crutcher MD, Grafton ST. Motor subcircuits mediating the control of movement extent and speed. Journal of neurophysiology. 2003;90(6):3958–3966. doi: 10.1152/jn.00323.2003 12954606 10.1152/jn.00323.2003

[45] Packard MG, Knowlton BJ. Learning and memory functions of the basal ganglia. Annual review of neuroscience. 2002;25(1):563–593. doi: 10.1146/annurev.neuro.25.112701.142937 12052921 10.1146/annurev.neuro.25.112701.142937

[46] Yamada H, Matsumoto N, Kimura M. Tonically active neurons in the primate caudate nucleus and putamen differentially encode instructed motivational outcomes of action. The Journal of neuroscience. 2004;24(14):3500–3510. doi: 10.1523/JNEUROSCI.0068-04.2004 15071097 10.1523/JNEUROSCI.0068-04.2004 PMC6729748

[47] Ramachandran VS. Encyclopedia of the Human Brain, Four-Volume Set. Academic Press; 2002.

[48] Van Dijk KR, Sabuncu MR, Buckner RL. The influence of head motion on intrinsic functional connectivity MRI. Neuroimage. 2012;59(1):431–438. doi: 10.1016/j.neuroimage.2011.07.044 21810475 10.1016/j.neuroimage.2011.07.044 PMC3683830

[49] Di Martino A, Fair DA, Kelly C, Satterthwaite TD, Castellanos FX, Thomason ME, et al Unraveling the miswired connectome: a developmental perspective. Neuron. 2014;83(6):1335–1353. doi: 10.1016/j.neuron.2014.08.050 25233316 10.1016/j.neuron.2014.08.050 PMC4169187

